# Validation and refinement of cropland data layer using a spatial-temporal decision tree algorithm

**DOI:** 10.1038/s41597-022-01169-w

**Published:** 2022-03-02

**Authors:** Li Lin, Liping Di, Chen Zhang, Liying Guo, Yahui Di, Hui Li, Anna Yang

**Affiliations:** 1 Center for Spatial Information Science and Systems, George Mason University, Fairfax, VA 22030 USA; 2 Department of Geography and Geoinformation Science, George Mason University, Fairfax, VA 22030 USA

**Keywords:** Agriculture, Geography

## Abstract

Space-based crop identification and acreage estimation have played a significant role in agricultural studies in recent years, due to the development of Remote Sensing technology. The Cropland Data Layer (CDL), which was developed by the U.S. Department of Agriculture (USDA), has been widely used in agricultural studies and achieved massive success in recent years. Although the CDL’s accuracy assessments report high overall accuracy on various crops classifications, misclassification is still common and easy to discern from visual inspection. This study is aimed to identify and resolve inaccurate crop classification in CDL. A decision tree method was employed to find questionable pixels and refine them with spatial and temporal crop information. The refined data was then evaluated with high-resolution satellite images and official acreage estimates from USDA. Two validation experiments were also developed to examine the data at both the pixel and county level. Data generated from this research was published online in two repositories, while both applications allow users to download the entire dataset at no cost.

## Abstract (summary)

**Table:** 

Measurement(s)	agricultural field
Technology Type(s)	decision tree
Sample Characteristic - Location	contiguous United States of America

## Background & Summary

Monitoring agricultural activities can provide valuable information to farmers, investors, and decision makers^1,2^, and crop identification and mapping is the fundamental basis to support agricultural studies^3–6^. Many applications and systems were created to classify land use land cover in recent decades due to developments in Remote Sensing and Geographic Information Science (GIS)^6–9^. Furthermore, the U.S. Department of Agriculture (USDA) has published the annual Cropland Data Layer (CDL), which covers the contiguous United States, since 2008^2,10–12^. The data was widely adopted in various agricultural studies after it was deployed to the CropScape system because of its state-of-the-art system architecture and adoption of geospatial Web services standards in a publicly accessible web environment^7,8,13–15^.

Since the production of CDL mainly relies on remote sensing datasets, criticisms have arisen in past years regarding the quality of CDL products^16,17^. To address this concern, the USDA National Agricultural Statistics Service (NASS) has published an accuracy assessment for the CDL since 2008^12^. The document contains detailed accuracy evaluations for each crop at the state level using ground truth validation data from other agencies (e.g., the USDA Farm Service Agency (FSA)). The annual CDL accuracy assessment reports provide valuable uncertainty measurements for scientific communities^18^. Although the confidence layers cannot be used directly to represent the accuracy of the CDL, the accuracy assessments are crucial in evaluating the quality of classification results for the CDL at the pixel level^16^. By combining the pixel-level confidence layer and state-level crop accuracy metrics, scientists can better understand the spatial variance of the classification accuracy of the CDL^16^. We acknowledge the usage and importance of both measurements; however, such an assessment cannot be utilized in this paper because the accuracy assessment and confidence layer are byproducts of the CDL production process, and this research is designed to conduct an independent review on CDL^17,19^.

Studies in recent years have demonstrated the possibility of refining existing crop mapping and predicting future or past planting information^9,20^. These studies utilized the historical archived remote sensing data and machine learning approaches, and results indicated the feasibility of improving the accuracy of CDL^9,20–23^. However, these methods usually only focus on a few crop types and are not scalable to border categories due to the shortage of training samples and labeled datasets^9^. In addition, although machine learning approaches could achieve high accuracy results, the training models used in machine learning are highly localized and often lose functionality when the study area is changed^24^. Thus, most of these studies concentrated on small regions.

This paper is aimed at evaluating CDL data and improving the accuracy of all crop types using spatial and temporal information with decision tree approaches. We developed a post-classifying algorithm for correcting false classified pixels in CDL using surrounding pixels and historical CDL information. The algorithm can be divided into two sections. The first section is devoted to identifying potential pixels of misclassification, and the second part of the algorithm predicts the correct classification for the pixels. More specifically, the proposed algorithm evaluated the classification types (crop or non-crop) of a pixel, the land use land cover types for the neighborhoods of a candidate pixel, historical appearance, and other reference data. The details of the proposed algorithm are further explained in the following sections.

**Fig. 1:**
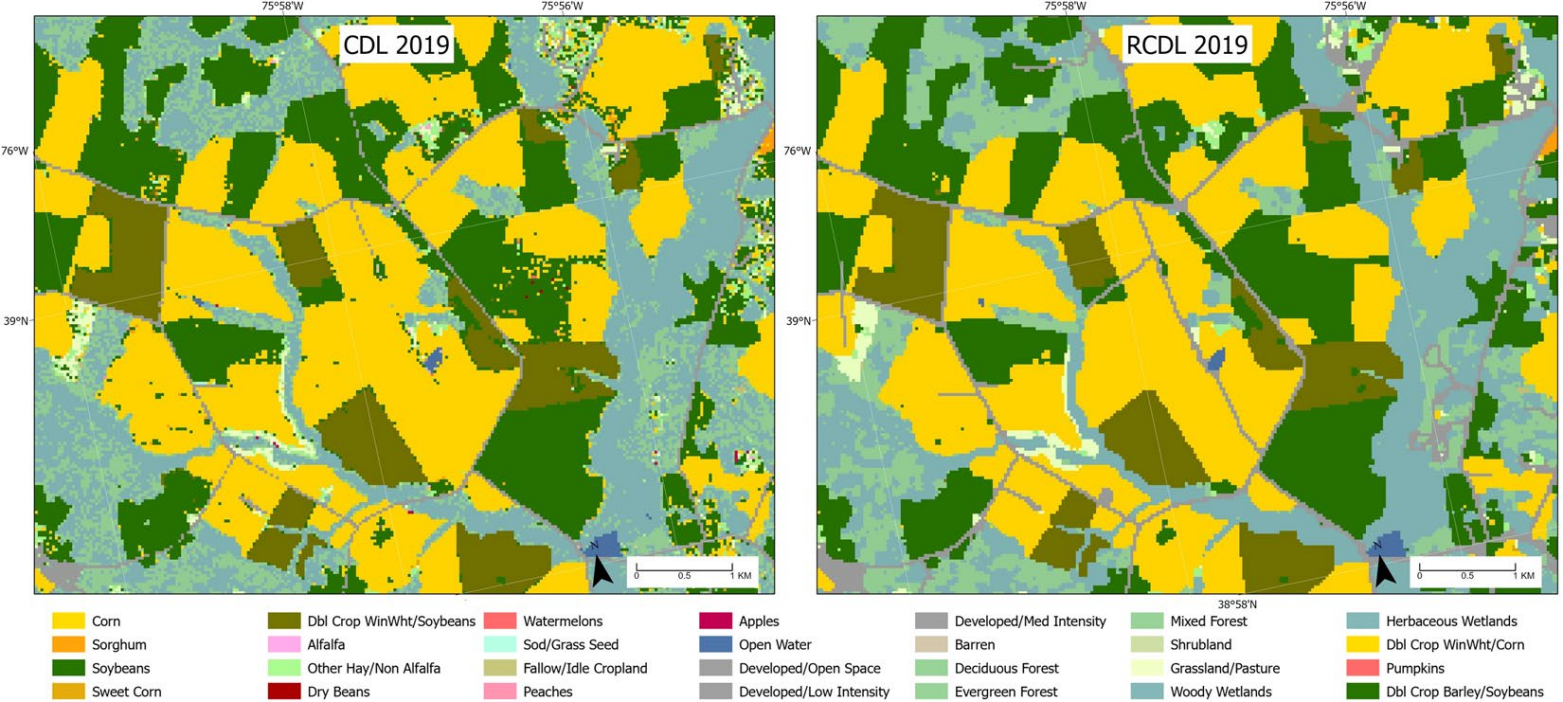
Comparing classification results for fields in Queen Anne’s County, Maryland, in 2019. The left image shows landcover data from USDA CDL, and the right image shows data from Refined CDL. The legend was adapted from the official CDL color map (https://nassgeodata.gmu.edu/CropScape).

When evaluating the proposed algorithm, two experiments were designed to validate the refined CDL (R-CDL) at both pixel-level and county-level. From visual comparisons, a significant reduction of false classification can be observed from our product (Fig. 1). Validation results have shown that the proposed algorithm has dramatically improved the classification accuracy with minor errors caused by mix-pixels at boundaries. We statistically compared the county level acreage estimates of two major crops (corn and soybean) for refined CDL (R-CDL) and CDL. We also calculated the acreage difference for both products with the official acreage estimates provided by NASS (https://quickstats.nass.usda.gov/). From the comparison experiment, we found that R-CDL has very similar R^2^ metrics with CDL compared to NASS acreage estimates, which indicates the R-CDL is reducing the classification noises. Still, it retains the high accuracy of acreage estimation for different crops at the county-level.

## Methods

### Identifying potential misclassifications in CDL

**Fig. 2:**
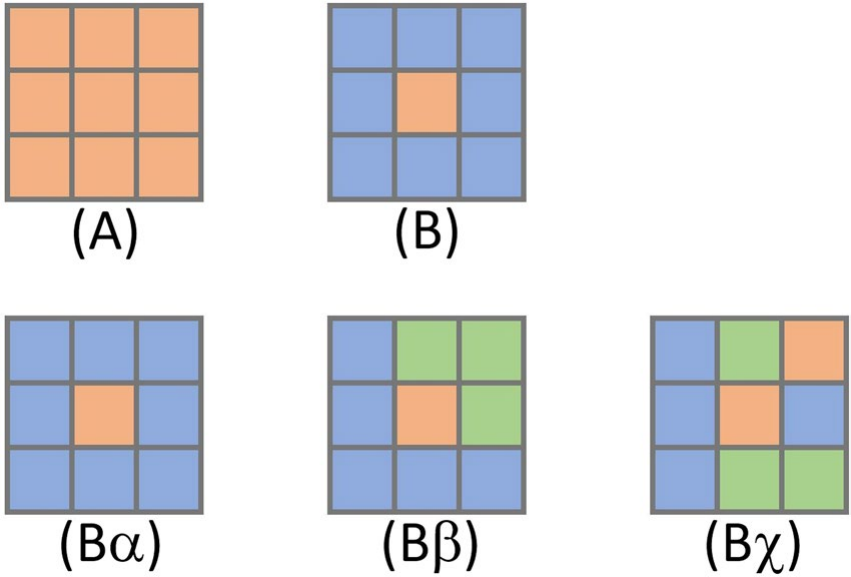
Separating all CLD pixels into different categories based on connecting neighborhoods (Pixels with different colors indicating different CDL codes).

As scholars suggested, most misclassifications in the CDL were identified at places with mixed land features (e.g., gaps between crop fields)^16^. For instance, a boundary pixel containing different crops may produce distinctive spectral signatures and lead to misclassification depending on the thresholds used in the production of CDL^10^. This error is mainly because the mapping unit (30 m) is too coarse to capture the variation within a pixel^9,16^. To identify false classifications, we proposed a spatial-temporal decision tree algorithm. Spatial-temporal patterns have been extracted, evaluated, and utilized in decision tree algorithms to solve scientific questions in the past studies^25–27^. The proposed decision tree algorithm first evaluates each pixel with their surrounding neighborhoods and puts them into different groups. The model uses all connected eight neighborhoods in this step, since linear features exist in the data. As shown in Fig. 2, all pixels can be grouped into two major categories: (A) a candidate pixel is the same as all eight connecting neighborhoods (all nine pixels are in the same class); and (B) the candidate is different from the surrounding connecting neighborhoods. Pixels in group A will be removed from our algorithm as it requires no further investigation. The second group (B) will be divided into three classes (the second row in Fig. 2): (Bα) isolate pixels, defined as when all eight connected pixels are in the same class; (Bβ) boundary pixel, defined as when there are more than four (but less than eight) same contiguous neighbor pixels; (Bχ) mix pixel, defined as all remaining pixels with no adjacent (more than four) neighborhoods.

Using spatial neighborhoods is an effective tool for spotting isolated and boundary pixels. However, it is not sufficient to conclude that these pixels are false classification. In fact, it is common to see farmlands contain non-agricultural landcover such as ponds or forests due to the nature of the earth. For this reason, historical CDL information is then extracted. The temporal information is then supplied to the decision tree model for providing historical information for each pixel. We decided to process CDL information from 2017 to 2020, as four years of data would produce sufficient output for nationwide validation, and since the CDL has provided national coverage since 2008, there would still be enough historical information for the proposed model. This research evaluated the nine years of CDL data before the study periods. For instance, CDLs from 2011 to 2019 were extracted for assessing the classification in 2020. The temporal ranges are the same for all four study years to ensure the outputs are generated under the same algorithm.

**Fig. 3:**
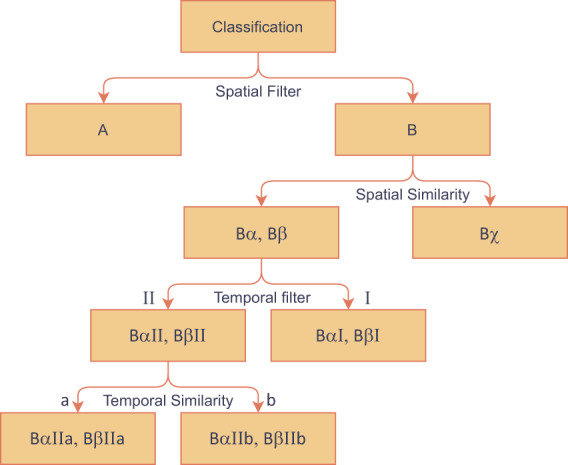
Proposed post-classification decision tree algorithm.

Based on all nine historical observations, dominating historical classifications for each pixel were identified. A dominating historical classification is defined as a pixel that was frequently classified as one category in the past nine years. Different thresholds were arbitrarily implemented for two different types of features: constant feature and non-constant feature. Constant features include natural elements such as bodies of water, forests and artificial features such as roads and buildings. Non-constant features are regions where the land use land cover is constantly changing, such as rotating planted croplands. Table 1 presents the CDL codes and class names for all constant classifications. Two arbitrary thresholds were defined: historical appearances needed to be higher or equal to seven out of nine years for constant features, and higher or equal to five out of nine years for regular features. The tie threshold for constant features is based on the hypothesis that the classification for a constant feature will not change. Based on this hypothesis, the historical classification will always be the same, while misclassifications are tolerable up to a few years. We have a loose threshold designed to accommodate changing/rotating land use, such as crop rotation. The value is set at five for two reasons: five out of nine is a good cutoff representation for rotating crops, and there is a possibility for multiple historical dominating classes for a pixel if the threshold drops below five (e.g., a pixel might have two historical dominating class if the threshold set at four). Candidate pixels can be grouped into two types based on whether there is a dominating historical classification (I); or no historical dominating class (II). For all pixels that have historical dominating classes, we further grouped them into the following: (a) the classification of the current year and historical dominating for a candidate pixel is the same; (b) the classification of the current year and historical dominating for a candidate pixel is different. The overview of the proposed decision tree model to identify false classification is illustrated using these symbols in Fig. 3.

**Table 1:** CDL codes and class names that were labeled as constant features in the algorithm^9,13^.

CDL Code	Classification Name
63	Forest
82	Developed
83	Water
87	Wetlands
111	Open Water Perennial
121	Developed/Open Space
122	Developed/Low Intensity
123	Developed/Med Intensity
124	Developed/High Intensity
141	Deciduous Forest
142	Evergreen Forest
143	Mixed Forest

### Post-classifying decision tree algorithm

**Fig. 4:**
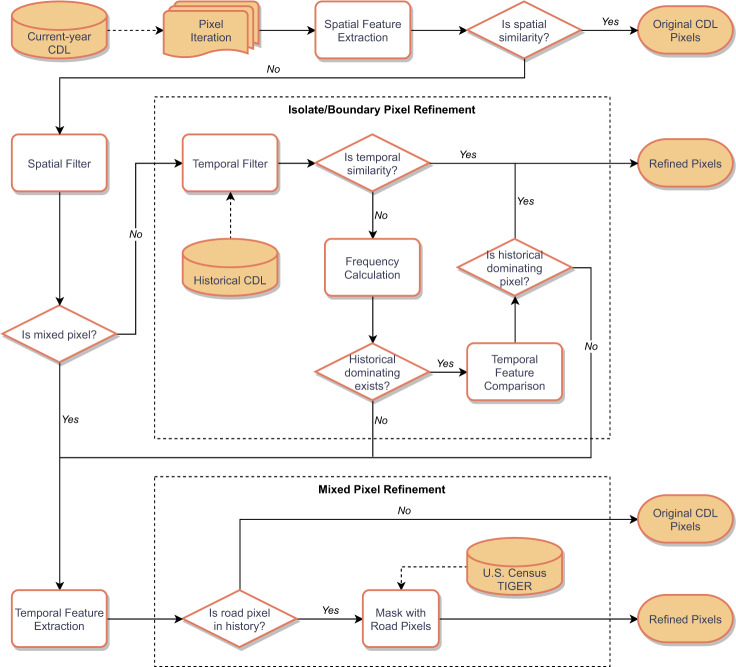
The refinement workflow of the proposed decision tree model.

The post-classifying decision tree algorithm proposed in this study adjusts misclassifications using spatial and temporal information. Details of the workflow are illustrated in Fig. 4. In this workflow, all pixels were classified into three types (good CDL pixels, isolate/boundary CDL pixels, and mixed CDL pixels) compared with connected cells using a spatial filter. We kept the majority class classification in system memory for isolate and boundary pixels and then compared this info with the historical classifications of candidate pixels. We extracted the temporal features and calculated the temporal frequencies for historical classifications. A pixel would then be refined when there was no conflict between their majority neighborhood and historical classification. When the candidate was different from its historical dominating or had no historic domination, it would be discarded from the post-classification process. In the algorithm’s post-processing, we utilized the annually updated road data acquired from the U.S. Census to reconstruct some misclassified road features^28^. We rasterized and resampled major roads to a 30-meter spatial resolution and masked them with the CDL. Then we checked all candidate pixels, which were not refined by the decision tree algorithm, to see if these locations have been classified as roads in the past. A post-classification operation was then performed based on the historical appearance of road features and the U.S. Census TIGER reference data. The refined pixels were then thrown back into the workflow and repeated for several runs until the output raster was stabilized (no further refinement). Some pixels only require one iteration, and others require up to four iterations before existing the workflow. The workflow ends when there is no further refinement for input pixels.

### Final maps

To create the Refined Cropland Data Layer (R-CDL), we first prepossessed the USDA CDL data in ESRI ArcGIS Pro (v2.7.0) software (https://www.esri.com/) to ensure all data was projected into the same spatial reference system. Equal area projection was used to preserve the accuracy of area calculation. We also rasterized and resampled (nearest neighborhood) road and rail shapefiles into 30 meter to match the resolution and extend it with CDL using ArcGIS Pro and the Python GDAL library (https://gdal.org/). The nearest neighborhood resampling method is ideal for discrete data (e.g., land use) since it does not create new values^29^. Next, we processed the historical CDL for extracting dominating classifications for each candidate pixel. Then all data was injected into our decision tree algorithm for identifying misclassification and making corrections. Four annual R-CDL maps were produced for the contiguous United States from the year 2017 to 2020.

### Evaluation metrics

To evaluate the R-CDL data, we designed the following two evaluation experiments. First, high-resolution images (Google Earth and Sentinel-1 images) were used as references for examining the accuracy of R-CDL classifications. Google Earth’s historical imagery archive is ideal for identifying fine objections such as small ponds or field houses^30,31^, and Sentinel-1 multi-band combinations provide a good reference when validating vegetation cover^32^. After overlaying the R-CDL with Google Earth and Sentinel images, visual inspections were conducted by remote sensing experts who manage and publish the CDL. We only validated the result of one year (2019) for two reasons: (1) all four years of data were processed with the same algorithm, so the result of a single year could represent the accuracy and performance for the proposed algorithm; (2) validating multiple-year would require acquiring more reference data which is a challenge for computer storage, especially with high-resolution images. The confusion metrics contain four classes: a false classified CDL pixel that was corrected in R-CDL, a false classified CDL pixel that was inaccurately refined in R-CDL, and third, a correct CDL pixel that was incorrectly refined in R-CDL. The last group includes correct CDL pixels that remained unchanged in R-CDL, which is not considered in our evaluation since the evaluation goal is to understand the accuracy of the refined pixels. The metrics are designed to delineate the success and errors of the refined results.

The second part of the evaluation was devoted to understanding the accuracy across classification categories in R-CDL. We compared the acreage data for corn and soybean in R-CDL and the CDL with the NASS estimate at the county-level, where NASS estimates are used as “ground truth” data. The official crop planting data has been released annually by USDA NASS since 1975. Multiple studies have concluded that the county level crop acreage data provided by NASS is one of the most reliable data sources for the entire United States^12,33^. The coefficient of determination (R^2^) was calculated for R-CDL with NASS estimates and for the CDL with NASS estimates. We compared the results to see (1) if the R-CDL had the same correlation to the “ground truth” (NASS estimates) compared to CDL; (2) if there were any unbalanced post-classification for different crop classes found by checking R^2^s for major crops (corn and soybean). In addition, using NASS estimates as a reference, we calculated the acreage differences for CDL and R-CDL with NASS estimates. Since NASS acreage estimation is often cited as one of the most accurate sources in terms of evaluating crop acreages^10,12,34,35^, we calculated and evaluated the planting area percentage variation for corn and soybean of R-CDL and CDL to NASS data.

## Data Records

The Refined Cropland Data Layer is available on In-season CROP Mapping Explorer System (iCrop), developed and operated by the Center for Spatial Information Science and Systems, George Mason University, at https://cloud.csiss.gmu.edu/icrop/. The iCrop system allows users to view, operate, and download R-CDL thru web-services at no cost.

The annual R-CDL data is also available for download from the Zenodo repository at 10.5281/zenodo.5567039^36^. Annual R-CDL layers are stored in.tif format, and other files in the folder store supportive information about the data. For example, georeferencing information is stored in the.tfw file. The folders of annual R-CDL layers are compressed and stored in.zip format to reduce the file size. The data can be viewed using various GIS software such as ESRI ArcGIS Pro (https://www.esri.com/) and QGIS (https://qgis.org/).

## Technical Validation

### Validating R-CDL post-classifications at pixel level using reference satellite observations

The first part of the technical validation was designed to provide a pixel-level cross-class assessment of the post-classification results. We first identified changing pixels by comparing CDL and R-CDL. Five hundred validation samples were then randomly selected among all refined pixels based on their geographic locations. Online-only Table 1 is the detailed matrix representing R-CDL classification corrections of sampled sites. From Online-only Table 1, we can observe that selected samples provided a balanced representation of various crop classes. It is worth noting that all non-cropland classes are grouped into one category (labeled as “NC”) to reduce the chart’s complexity. In total, 149 sample pixels were non-cropland to non-cropland, and 351 pixels contained at least one cropland classification. The confusion matrix of the post-classification accuracy of the validation sample is shown in Table 2. The overall accuracy of the post-classification result is about 80%, and if we do not consider true negatives, the accuracy is over 90%, indicating that the proposed algorithm yields a huge improvement in crop mapping. True negatives are misclassified CDL pixels that were refined in R-CDL, but still have a negative result. True negatives are mainly occupied by mix-pixels, and approaches to resolve mix-pixel misclassification have been discussed in the final section of this paper.

**Table 2:** Confusion matrix of the post-classification result of the validation set.

Classification result		R-CDL	
		correct	incorrect
CDL	correct	N/A	35
	incorrect	394	71

### Comparing spatial and temporal agreement of crop areas for R-CDL with CDL statistics and NASS estimates

**Fig. 5:**
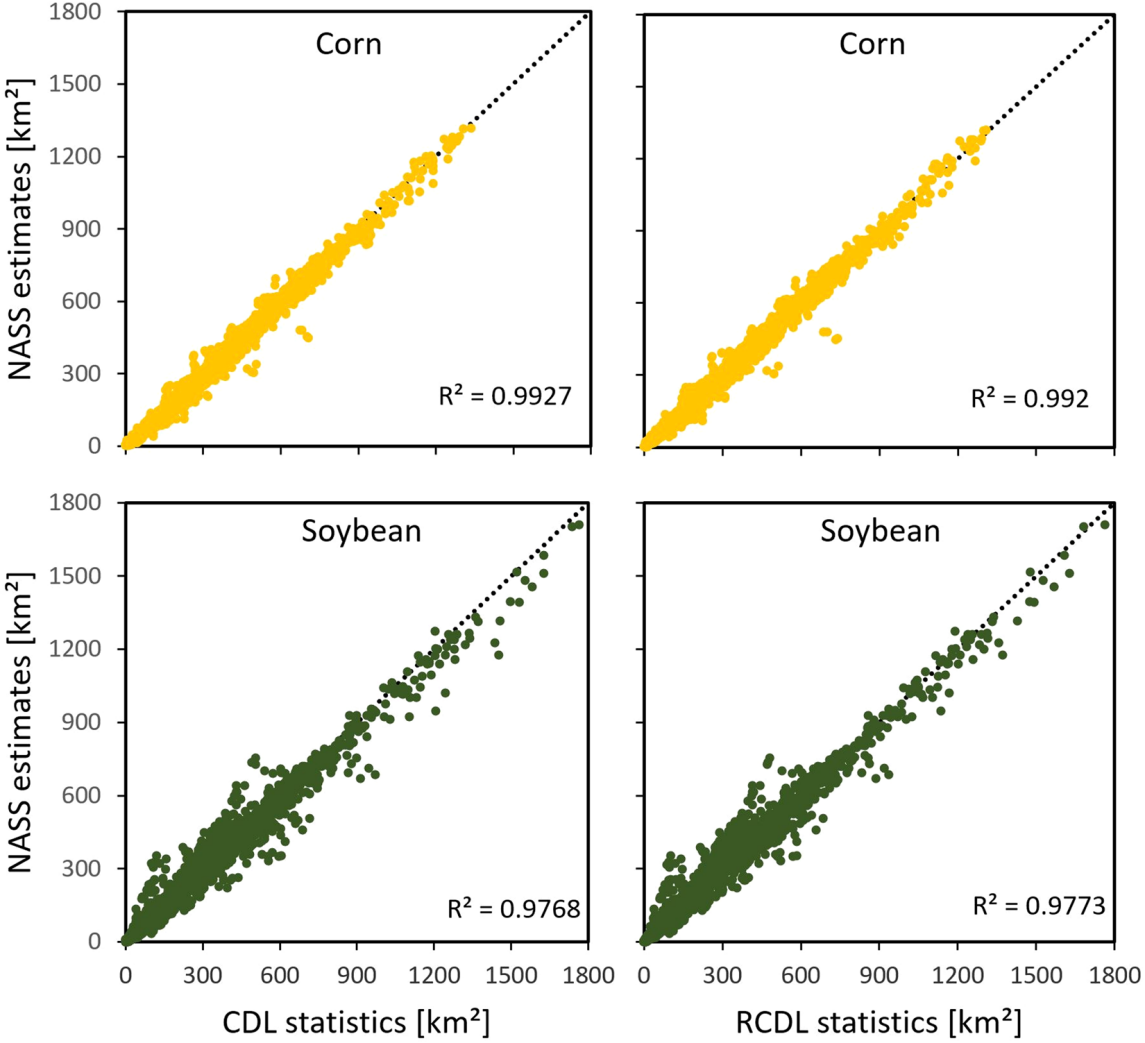
Comparing NASS county-level planted area with CDL or R-CDL statistics for corn and soybean from 2017–2020. Correlation is measured by the coefficient of determination, R^2^. Depending on the data availability of NASS estimates, 838 counties were calculated for corn, and 836 counties were calculated for soybean.

In addition to the pixel-level accuracy assessment, the research also validated the acreage accuracy at the county-level by comparing CDL and R-CDL to NASS acreage estimates. Fig. 5 presents the county-level planting area agreement between NASS estimate and CDL or R-CDL for corn and soybean from 2017 to 2020. Since NASS does not assess planting information for all counties in the United States, it is not possible to count all counties in the evaluation. However, counties with NASS estimates for 2017 to 2020 were all included in our assessment. 838 and 836 counties were used for planting area comparison for corn and soybean, respectively. The result has shown that the R^2^ for R-CDL-NASS is very high in acreage estimation at the county-level. The high accuracy indicates that the planting area for R-CDL is statistically correlated with acreage estimation from NASS. In addition, R^2^s for corn and soybeans between our algorithm and NASS are almost the same compared to CDL-NASS.

To further break down the crop planting statistics, we divided the comparison by study years. Table 3 and Table 4 show the average distance (in percentage) from the CDL and R-CDL to NASS estimates, where positive distance means it is higher than NASS estimates, and a negative distance means it is lower. On average, county-level acreage for R-CDL is closer to NASS estimates than for the CDL. In other words, our algorithm improved the acreage statistics for corn estimates for all four study years and improved three out of four years for soybean planting area estimates. In addition, the statistics from Table 4 imply that the corn acreage for R-CDL is near identical to NASS estimates, especially for the past three years (average net difference less than 1%). On the other hand, soybean acreage has a bigger gap (about 3%) between R-CDL and NASS estimates. The result suggests two possibilities: (1) the proposed method is more effective on correct corn misclassification than soybean, or (2) NASS overestimated the acreage of soybean for the past years.

**Table 3:** Average percentage difference for corn planting areas at county level from NASS to CDL and R-CDL in different years.

Corn	2017	2018	2019	2020
CDL	4.2%	6.7%	6.7%	9.6%
R-CDL	−2.1%	−0.5%	−1.1%	0.6%

**Table 4:** Average percentage difference for soybean planting areas at the county-level from NASS to CDL and R-CDL in different years.

Soybean	2017	2018	2019	2020
CDL	2.7%	3.9%	5.1%	4.1%
R-CDL	−3.6%	−3.0%	−2.2%	−3.5%

A few conclusions were drawn from the two technical validation experiments. The pixel-level validation and county-level planting area comparison lead us to conclude that our algorithm is highly effective in removing spark misclassifications in CDL while causing minimal damages to correct classifications. Although the validation sample size is not broad enough, the overall improvement of classification accuracy is dramatic. It is worth noting that, in the second experiment, NASS acreage estimates are not ground truth data; however, this estimate is the closest acreage estimation for croplands in the United States. Thus, the second experiment quantitatively demonstrated that our algorithm has slightly improved the acreage estimation statistically by correcting these spark pixels in CDL.

### Limitation and future work

In this study, a spatial-temporal decision tree algorithm was developed to identify and refine misclassification in the CDL. Although the algorithm successfully identified and corrected spark errors, there are a few limitations in the decision tree algorithm and R-CDL. First of all, the validation of this research relied on other satellite images and NASS acreage estimates. These data sources are generally reliable, but they are not ground observations, and thus could contain errors that could contribute to uncertainties in our product. Although this research utilized them as references since historical ground truth data does not exist or is classified, as technologies improve, we may have better data sources for future validation purposes. Second, the algorithm has limited functionality in correcting mix pixels since their historical and neighborhood classification might not provide valuable information based on our model. Most misclassifications in refined CDL are from mix pixels according to the observation during technical validation. The ideal solution to this issue is utilizing the Common Land Unit (CLU) to precisely identify the boundaries of different land covers. However, this data is no longer accessible to the public and only available to certain USDA agencies^37,38^. Alternatively, other methods such as machine learning could be utilized for refining these pixels. Future studies are encouraged to use this novel technology for evaluating mix pixels in our product. Third, the evaluation experiments established in this study are broad and generalized. On one hand, the metrics captured overall accuracy and thus provided a quick assessment for our algorithm; on the other hand, more thoughtful and detailed evaluations are needed to better understand the spatial-temporal variation for our data compared to CDL. Lastly, similar to the previous statement, this research mainly focuses on crop classification correction, and the algorithm’s performance on non-agricultural fields is not evaluated, especially for areas that experienced rapid changes, such as expanding/shrinking cities^24^. The accuracy for these areas may be lower and would require future validations.

## Usage Notes

Compared with Cropland Data Layer, the results of the refining algorithm developed in this research have better accuracy according to our validation. However, although comprehensive validation experiments were conducted for delineating the accuracy for a few crops, we did not validate all pixels and all classification categories. We recommend users read the limitations section and consider validating the product before using this data for their studies.

## Data Availability

The scripts used to generate the R-CDL dataset are available in this GitHub repository: https://github.com/llin-csiss/RCDL.

## Online-only Table

**Online-only Table 1:** Matrix of validation samples and their classifications in CDL and R-CDL, 267 respectively.

	RCDL	Corn	Cotton	Sorghum	Soybeans	Peanuts	Barley	Spring Wheat	Winter Wheat	Dbl Crop WinWht/Soybeans	Oats	Millet	Canola	Alfalfa	Sugarbeets	Dry Beans	Sugarcane	Peas	Christmas Trees	Dbl Crop WinWht/Corn	Blueberries	Non-Crop	Total
CDL		1	2	4	5	10	21	23	24	26	28	29	31	36	41	42	45	53	70	225	242	NC	
Corn	1	—	0	0	14	0	0	0	2	0	0	1	0	0	0	0	0	0	0	0	0	48	65
Cotton	2	0	—	1	2	2	0	0	0	0	0	0	0	0	0	0	0	0	0	0	0	6	11
Rice	3	0	0	0	1	0	0	0	0	0	0	0	0	0	0	0	0	0	0	0	0	0	1
Sorghum	4	1	0	—	2	0	0	0	0	0	1	0	0	1	0	0	0	0	0	0	0	8	13
Soybeans	5	20	2	0	—	0	1	0	1	1	0	0	0	0	0	0	1	0	0	1	0	58	85
Sunflower	6	0	0	0	0	0	0	0	0	0	0	0	0	0	0	1	0	0	0	0	0	1	2
Peanuts	10	0	0	0	0	—	0	0	0	0	0	0	0	0	0	0	0	0	0	0	0	1	1
Barley	21	0	0	0	0	0	—	1	0	0	0	0	0	0	0	0	0	0	0	0	0	1	2
Spring Wheat	23	1	0	0	0	0	2	—	0	0	0	0	1	0	0	0	0	0	0	0	0	8	12
Winter Wheat	24	1	0	0	1	0	0	0	—	2	0	0	0	1	0	0	0	0	0	0	0	13	18
Dbl Crop WinWht/Soybeans	26	0	0	0	1	0	0	0	1	—	0	0	0	0	0	0	0	0	0	0	0	2	4
Rye	27	0	0	0	0	0	0	0	0	0	0	0	0	0	0	0	0	0	0	0	0	2	2
Oats	28	1	0	0	1	0	0	0	1	0	—	0	0	0	0	0	0	0	0	0	0	3	6
Millet	29	0	0	0	0	0	0	0	0	0	0	—	0	0	0	0	0	0	0	0	1	0	1
Canola	31	0	0	0	0	0	0	0	0	0	0	0	—	0	0	0	0	1	0	0	0	1	2
Flaxseed	32	0	0	0	1	0	0	0	0	0	0	0	0	0	0	0	0	0	0	0	0	0	1
Alfalfa	36	4	0	0	4	0	0	1	0	0	0	0	0	—	0	0	0	0	0	0	0	19	28
Sugarbeets	41	2	0	0	0	0	0	0	0	0	0	0	0	0	—	0	0	0	0	0	0	1	3
Dry Beans	42	0	0	0	1	0	0	0	0	0	0	0	0	0	1	—	0	0	0	0	0	0	2
Other Crops	44	0	0	0	0	0	0	0	0	0	0	0	0	0	0	0	0	0	0	0	0	1	1
Sugarcane	45	0	0	0	0	0	0	0	0	0	0	0	0	0	0	0	—	0	0	0	0	1	1
Onions	49	0	0	0	0	0	0	0	0	0	0	0	0	0	0	0	0	0	0	0	0	1	1
Cucumbers	50	0	0	0	0	0	0	0	0	0	0	0	0	0	0	0	0	0	0	0	0	1	1
Tomatoes	54	0	0	0	0	0	0	0	0	0	0	0	0	0	0	0	0	0	0	0	0	1	1
Herbs	57	0	0	0	0	0	0	0	0	0	0	0	0	0	0	0	0	0	0	0	0	1	1
Sod/Grass Seed	59	0	0	0	1	0	0	0	0	0	0	0	0	0	0	0	0	0	0	0	0	1	2
Peaches	67	0	0	0	0	0	0	0	0	0	0	0	0	0	0	0	0	0	0	0	0	1	1
Grapes	69	0	0	0	0	0	0	0	0	0	0	0	0	0	0	0	0	0	0	0	0	1	1
Pecans	74	0	0	0	0	0	0	0	0	0	0	0	0	0	0	0	0	0	0	0	0	1	1
Non-Crop	NC	30	3	2	27	0	0	5	5	1	0	0	0	7	0	0	0	0	1	0	0	—	81
Total		60	5	3	56	2	3	7	10	4	1	1	1	9	1	1	1	1	1	1	1	182	351
